# Supplementary material for: Analysis of residential satisfaction: An empirical evidence from neighbouring communities of Rohingya camps in Cox’s Bazar, Bangladesh
Source: PLoS One. 2021 Apr 29;16(4):e0250838. doi: 10.1371/journal.pone.0250838 (PMC8084219; doi:10.1371/journal.pone.0250838)
Supplement: S1 Table — (DOCX) [file pone.0250838.s001.docx]

**S1 Table. Descriptive statistics of the variables**

| **Variables** | **Unit of measurement** | **Obs** | **Mean** | **Std. Dev.** | **Min** | **Max** |
| --- | --- | --- | --- | --- | --- | --- |
| Age | Years | 151 | 42.543 | 12.665 | 19 | 75 |
| Gender | Male =1, Otherwise=0 | 151 | .695 | .462 | 0 | 1 |
| **Marital status:** | Percentage |  |  |  |  |  |
| - Married | ,, | 151 | .815 | .39 | 0 | 1 |
| - Unmarried | ,, | 151 | .126 | .333 | 0 | 1 |
| - Divorced | ,, | 151 | .033 | .18 | 0 | 1 |
| - Wiodow | ,, | 151 | .026 | .161 | 0 | 1 |
| Household Size | Number | 151 | 5.132 | 1.81 | 1 | 13 |
| Education | Years | 151 | 5.642 | 4.417 | 0 | 17 |
| **Religion:** | Percentage |  |  |  |  |  |
| - Muslim | ,, | 151 | .616 | .488 | 0 | 1 |
| - Hindu | ,, | 151 | .139 | .347 | 0 | 1 |
| - Buddha | ,, | 151 | .245 | .432 | 0 | 1 |
| Residential status | *By birth=1, Otherwise=0* | 151 | .881 | .325 | 0 | 1 |
| Size of the floor | *Square feet* | 151 | 513.046 | 982.556 | 32 | 11250 |
| Distance from camp area | *Kilometer* | 151 | 5.778 | 9.984 | .015 | 30.2 |
| Children | Number | 151 | 2.053 | 1.331 | 0 | 7 |
| Sick persons | Number | 151 | .596 | .723 | 0 | 3 |
| Employment type | *Formal=1, Otherwise=0* | 151 | .146 | .354 | 0 | 1 |
| Working wives | *Yes=1, No=0* | 151 | .146 | .354 | 0 | 1 |
| Property right | *Yes=1, No=0* | 151 | .94 | .238 | 0 | 1 |
| Monthly income | BDT | 151 | 13536.424 | 11583.336 | 2000 | 100000 |
| Monthly expenditure | BDT | 151 | 10670.861 | 8032.447 | 1800 | 70000 |
| **Work opportunities:** | Percentage |  |  |  |  |  |
| - Increased | ,, | 151 | .126 | .333 | 0 | 1 |
| - Same | ,, | 151 | .219 | .415 | 0 | 1 |
| - Decreased | ,, | 151 | .656 | .477 | 0 | 1 |
| Mobility experiences | Percentage | 151 | .086 | .281 | 0 | 1 |
| **Residential satisfactions issues:** |  |  |  |  |  |  |
| - Security of social crime | 5 Points likert scale | 151 | 2.119 | 1.321 | 1 | 5 |
| - Cleanliness | ,, | 151 | 2.298 | 1.274 | 1 | 5 |
| - Crowdedness | ,, | 151 | 2.152 | 1.264 | 1 | 5 |
| - Social bonding | ,, | 151 | 3.391 | 1.107 | 1 | 5 |
| - Pollution | ,, | 151 | 2.073 | 1.178 | 1 | 5 |
| - Water supply | ,, | 151 | 2.417 | 1.235 | 1 | 5 |
| - Garbage management | ,, | 151 | 2.272 | 1.166 | 1 | 5 |
| - Density of housing | ,, | 151 | 2.172 | 1.171 | 1 | 5 |
| - Greenery | ,, | 151 | 2.099 | 1.38 | 1 | 5 |
| - Education facilities | ,, | 151 | 2.325 | 1.299 | 1 | 5 |
| - Health services | ,, | 151 | 2.603 | 1.276 | 1 | 5 |
| - Public transport | ,, | 151 | 1.603 | 1.114 | 1 | 5 |
| - Access to recreational | ,, | 151 | 2.007 | 1.262 | 1 | 5 |
| - Relief intervention | ,, | 151 | 2.609 | .848 | 1 | 5 |
| - Union Parishad services | ,, | 151 | 2.278 | 1.184 | 1 | 5 |
| - Size of the floor | ,, | 151 | 3.146 | 1.246 | 1 | 5 |
| - Floor level | ,, | 151 | 3.497 | 1.227 | 1 | 5 |
| - Kitchen | ,, | 151 | 2.874 | 1.207 | 1 | 5 |
| - Dinning space | ,, | 151 | 3 | 1.217 | 1 | 5 |
| - Bedroom | ,, | 151 | 3.04 | 1.119 | 1 | 5 |
| - Toilet | ,, | 151 | 2.861 | 1.414 | 1 | 5 |
| - Quality of the dwelling | ,, | 151 | 2.815 | 1.293 | 1 | 5 |
| **Satisfaction with** |  |  |  |  |  |  |
| - Social environment | Percentage | 151 | 48.168 | 20.329 | 20 | 100 |
| - Neighbourhood environment | ,, | 151 | 43.62 | 23.091 | 20 | 100 |
| - Public services and facilities | ,, | 151 | 44.746 | 17.224 | 20 | 83.333 |
| - Dwelling unit | ,, | 151 | 60.662 | 18.041 | 20 | 94.286 |
| Overall residential satisfaction | ,, | 151 | 50.59 | 14.62 | 22.727 | 85.455 |
